# Supplementary material for: Novel Multicomponent Digital Care Assistant and Support Program for People After Stroke or Transient Ischaemic Attack: A Pilot Feasibility Study
Source: Sensors (Basel). 2024 Nov 13;24(22):7253. doi: 10.3390/s24227253 (PMC11598503; doi:10.3390/s24227253)
Supplement: Supplementary file 1 [file sensors-24-07253-s001.zip › sensors-3277731-supplementary.pdf]

This is a multimedia appendix for the full manuscript titled "**A novel multicomponent digital Care Assistant and support Program for people after Stroke or transient ischaemic attack (CAPS): a pilot feasibility study".**

Table S1. Secondary Prevention Goal Attainment at 12-week follow-up

**Type of goals**	**Total number of goals, n (%)**	**Goal attained, n (%), N = 22**			**Goal not attained, n (%), N = 30**		
		**A lot more**	**A little more**	**As expected**	**Partially achieved**	**No change**	**Got worse**
Total	52 (100)	10 (19)	3 (6)	9 (17)	15 (29)	13 (25)	2 (4)
Exercising	21 (40)	4 (19)	1 (5)	4 (19)	4 (19)	7 (33)	1 (4)
Losing or gaining weight	12 (23)	2 (17)	0	1 (8)	5 (42)	4 (33)	0
Controlling blood pressure	5 (10)	1 (20)	0	2 (40)	2 (40)	0	0
Reducing alcohol consumption	4 (8)	0	2 (50)	1 (25)	1 (25)	0	0
Eating well	3 (6)	0	0	0	1 (33)	2 (67)	0
Feeling less depressed or anxious	3 (6)	3 (100)	0	0	0	0	0
Managing diabetes	2 (4)	0	0	0	1 (50)	0	1 (50)
Managing cholesterol	1 (2)	0	0	0	1 (100)	0	0
Managing atrial fibrillation	1 (2)	0	0	1(100)	0	0	0
